# Supplementary material for: Are carbon emissions trading and green financial instruments synergistic? -Comprehensive quantitative research based on content analysis
Source: PLoS One. 2024 Mar 7;19(3):e0298601. doi: 10.1371/journal.pone.0298601 (PMC10919601; doi:10.1371/journal.pone.0298601)
Supplement: S1 Data — (ZIP) [file pone.0298601.s001.zip › Data/地级市Z_F工作报告.zip!/╣π╢1⁄2/╟σ╘╢╩╨/└┤╫╘╣1⁄2╓┌║┼í╛┬φ┐╦╩2╛▌═°í┐_2006.docx]

各位代表：

我代表市人民政府向大会作政府工作报告，请予审议，并请政协各位委员和其他列席人员提出意见。

一、“十五”时期工作回顾

“十五”时期是极不平凡的五年，是我市经济社会发展取得巨大成就的五年。在省委、省政府和市委的正确领导下，在市人民代表大会及其常务委员会和市政协的监督支持下，市政府坚持以邓小平理论和“三个代表”重要思想为指导，全面落实科学发展观，认真贯彻落实党的十六大和省委九届全会以来的方针政策，围绕“三年打基础，五年上台阶，十年翻两番，二十年全面建成小康”的总目标，大力实施“三化一园”发展战略，采取了各种切实有效的工作措施，成功战胜非典型肺炎疫情、高致病性禽流感和严重干旱、洪涝等自然灾害，有效解决了经济发展中的交通、土地、电力、资金等“瓶颈”制约，顺利实现了市人大四届三次会议确定的2005年各项预期目标，圆满完成了市人大三届四次会议确定的“十五”计划各项任务。

“十五”时期，我市经济社会发展实现了历史性的跨越，人民生活达到总体小康水平，城乡面貌发生显著变化，社会各项事业全面进步，国民经济持续快速健康发展。在全省公布的八项主要经济指标中，我们在2004年取得了四项全省增长第一的基础上，2005年又取得了GDP、规模工业总产值、规模工业增加值、社会消费品零售总额、外贸出口总额、实际利用外资金额、固定资产投资总额等七项指标增长全省第一，地方财政一般预算收入增长全省第二的历史最好成绩，实现了主要经济指标增长“双连冠”目标。地区综合经济实力和竞争力显著增强，创造了令人瞩目的“清远现象”。

（一）经济发展实现大跨越。

经济实力显著增强。2005年实现GDP 315.7亿元，比上年增长23.3%，高出全省平均增幅10.8个百分点，比2000年翻一番，五年年均增长14.5%。经济总量在全省21个地级以上市中排列第15位，比2000年的19位前移4位，成为全省经济总量跃升最快的市。部分指标已位居全省中游水平，其中，固定资产投资总额列第8位、实际利用外资金额列第11位、规模以上工业总产值和增加值均列第13位。人均GDP突破1000美元大关，我市经济已进入一个加速发展的黄金时期。

经济结构明显改善。实现了工农业比例和粮经比例“两大掉头”。2005年工业产值占工农业产值比重达到80%，比2000年上升30个百分点；粮食播种面积与经济作物种植面积比例由2000年的54：46调整到2005年的47：53。第二、三产业发展迅猛，三次产业结构由2000年的39：26：35，发展到2005年的23：37：40。

工业生产高速发展。2005年，全部工业总产值达到402亿元，比上年增长68.9%，高出全省平均增幅57.1个百分点，比2000年翻两番，五年年均增长28.9%。特别是近两年，规模以上工业总产值均保持60%以上的增速，成为全省工业发展最迅猛的地区。2005年，全部工业实现增加值92.6亿元，比上年增长55%，高出全省平均增幅38个百分点，五年年均增长24.4%。我市确定的十大支柱产业规模迅速扩大，总产值达到232亿元，增长2.55倍，占全部规模以上工业产值的66%，比“九五”期末提高9个百分点。

经济效益不断好转。企业盈利能力不断增强，经济增长质量明显提高。“十五”时期规模以上工业累计盈利18.5亿元，扭转了“九五”时期累计亏损6.2亿元的局面。2005年规模以上工业实现利润总额7.7亿元，比上年增长98.0%，五年年均增长27.3%；实现利税16.6亿元，比上年增长60.3%，五年年均增长21.4%。

农业生产稳步增长。农村基础设施建设不断加强，农业综合生产能力持续提升，抗灾害、御疫情能力进一步增强。农业规模化、产业化经营水平明显提高。“八个五”工程建设进展顺利。成功举办了第一、二届中国华南博会，引进了一批农业龙头企业，促进了农村产业化经营组织迅速发展，2005年底，全市具有一定规模的农业产业化经营组织126家，比“九五”期末增加87家。其中，农业龙头企业88家，增加52家；年销售收入超亿元的企业10家。建立省级农业标准化示范区7个，75家生产单位通过无公害农产品产地认定，105个产品通过无公害认证。市级28个“一乡一品”项目生产规模达15.56万亩，增长3.7倍。农业生产持续稳定发展，2005年实现农林牧渔业总产值107.8亿元，比上年增长4.0%，五年年均增长4.0%。

第三产业发展迅猛。旅游业实现跨越式发展。2005年全市旅游区（点）发展到60处，比2000年增加了51处；打造了清远温泉、清远漂流、清远山水、清远奇洞、清远风情等五大旅游品牌，“五游俱全，唯有清远”的地区旅游形象不断提升，多条旅游热线竞争力日益增强。全市游客达到1005万人次，旅游收入32.63亿元，分别比上年增长33.1%和42.8%，五年年均增长28.7%和30.5%。房地产业高速发展，碧桂园假日半岛一、二期、金碧湾花园、金海湾豪庭、城市广场等高档次楼盘相继建成，已建成或在建五星级标准酒店多家，已成为广东新的房地产投资热点之一，2005年清远房地产价格升幅全省第一。完善商贸流通设施，培育消费热点，城乡消费旺盛。2005年实现社会消费品零售总额129.8亿元，比上年增长19.5%，五年年均增长12.0%。第三产业实现增加值126.9亿元，比上年增长18.3%，五年年均增长13.5%。对外贸易强劲增长，2005年外贸出口总额9.3亿美元，比上年增长77.0%，高出全省平均增幅52.7个百分点，五年年均增长30.9%。

民营经济发展强劲。鼓励民营经济发展优惠政策得到落实。2005年全市注册个体工商经营户6.8万户，注册资金21.82亿元，比2000年增长1.4倍；民营企业3558家，注册资金77.6亿元，分别增长2.02倍和5.14倍。民营经济投资95.1亿元，占全市固定资产投资总额的42%。全市规模以上民营工业企业160家，增长2.54倍，部分民营企业产值已达到10亿元以上。2005年，全市民营企业实现工业总产值118.8亿元，比上年增长1.1倍，五年年均增长50.0%，已成为推动我市经济发展的重要力量。

财税收入快速增长。2005年，来源于清远的财税总收入32.9亿元，税收总收入30.7亿元，地方财政一般预算收入13.25亿元，分别比上年增长31.1%、34.2%和39.1%，五年年均增长33.2%、34.1%和24.0%。“十五”时期实现地方财政一般预算收入42.9亿元，是“九五”时期的2.33倍。

物价金融形势稳定。总体物价水平保持稳定，宏观经济处在高增长低通胀的良好状态。五年来居民消费价格总水平上涨1.7%。2005年末，全市金融机构各项存款余额386.2亿元，比上年末增长15.3%，五年年均增长13.3%；各项贷款余额175.1亿元，比上年末增长4.1%，五年年均增长3.9%。

（二）招商引资取得丰硕成果。

“十五”期间，我市把招商引资作为经济工作的重中之重，充分抓住国际、国内经济结构调整和产业升级转移的大好时机，合理利用土地资源，创办工业园区，建立和完善优惠政策及奖励政策，着力营造“洼地效应”，对外商投资吸引力显著增强。通过下移招商引资主体，创新招商引资方法，大力开展招商引资工作，取得了突破性的成果，呈现出引进项目越来越大、投资金额越来越多、技术含量越来越高、地方财力贡献越来越大的喜人局面。尤其是2003年以来，全市共引进项目1680多个，计划总投资1220多亿元,实际已投入280多亿元。其中投产项目870多个，在建项目460多个，筹备项目340多个。2005年，全市共引进项目712个，比上年增加78个；计划总投资490.5亿元，增长43.0%；累计实际投入资金84.65亿元，增长2.28倍。其中投产项目233个，在建项目246个，筹备项目233个。“十五”期间，全市累计实际利用外资金额6.86亿美元。海螺水泥、台湾水泥、东鹏陶瓷、新中源陶瓷、蒙娜丽莎陶瓷、圣力钢铁、大金源不锈钢、双汇食品、金德管业、毅力电子、豪美铝材、广硕鞋业等一批著名大企业和大项目先后落户我市，对我市经济发展起到了良好推动作用。

（三）基础设施和市政建设迈上新台阶。

2005年,全市固定资产投资完成225.9亿元，比上年增长41.6%，高出全省平均增幅27.7个百分点。“十五”时期累计完成固定资产投资601亿元，比建国以来至2000年51年间的投资总和还多116亿元。

基础设施显著改善。基础设施建设累计完成投资193亿元，其中交通设施投资75亿元。完成了广清高速公路北段、英佛一级公路等第一个 “八路一桥”工程；第二个“八路一桥”工程全面推进，其中大燕河至银盏一级公路加铺沥青改造工程、市区环城北段一级公路扩建工程一期清新大桥及引道工程已完工；完成县乡公路建设与改造2132公里，在全省山区市率先实现县（市、区）通镇公路全部硬底化目标。2005年末，全市公路通车里程达到10854.6公里，五年新增1614.6公里，其中高速公路114.9公里；每平方公里公路密度从2000年的48.2公里提高到56.7公里。水利设施累计投入23.03亿元。完成了一批城乡水利防灾减灾工程、农村饮水工程、水库除险加固工程等重点项目，加固江河堤围116.15公里，新增除涝面积8.2万亩，解决农村饮水困难人口36.35万人，水利防灾减灾能力显著提高。能源设施投资76.1亿元。完成了粤连火电厂二期和阳山火电厂改造等一批水、火电工程项目。2005年末全市发电装机容量达到195万千瓦，比“九五”期末增加70万千瓦。完成30项110千伏以上输变电工程以及农网改造工程，新增220千伏输电线路528公里， 110千伏输电线路429公里；新增主变容量140万千伏安。通信设施建设成效显著，已达到全省平均水平。2005年，全市互联网用户8.48万户，比2000年增长3倍；电话机用户197.5，平均两人有一部，其中，移动电话用户134.3万户，增长5.5倍。

城市化进程明显加快。进一步加强城市中轴线、水轴线、主干道路和公共设施的规划建设。市区完成了广清大道、清远大道、凤城大桥、凤翔大道、桥北路和桥南路等骨干项目的建设与改造，大市区框架初步奠定，服务功能配套不断完善，城市形象日益提升。英德、连州、佛冈、清新、阳山等县城（市区）的中轴线建设已呈现雏形，城镇建设、改造步伐加快，配套设施进一步完善，中心城镇服务能力大大增强。各地城镇道路美化、绿化、亮化工作进一步加强，其中，市区绿地面积达735.3公顷，绿化覆盖率32.58%，人均绿地面积5.41平方米。全市城市化水平达到28.8%，比2000年提高8.7个百分点。

（四）改革创新取得显著成效。

国有企业改革稳步推进，各县（市、区）完成了90%以上的国有企业改革任务，市属预算内国有企业基本退出竞争领域。国有资产管理迈出新步伐，成立了清远市人民政府国有资产监督管理委员会，对国有资产统一实施监督管理。政府机构改革顺利完成，全市精减行政机构6%，精减行政编制25%；行政审批制度改革进展顺利，市政府原有审批、核准事项由567项减少到192项，减幅达66.1%。行政区域改革稳步推进，撤销了飞来峡管理区，完成了英红华侨管理区的撤区建镇工作，全市乡镇由143个减少到80个，共撤并乡镇63个，撤并率达到44%，比省下达的15%的目标高出29个百分点。卫生、教育、交通资源整合卓有成效。“十五”期间新增省一级中小学12所，新增优质中小学学位13.38万个；市中心区域卫生资源通过有效整合，效益明显好转；撤并中心区域路桥收费站，实行年票制。农村税费改革全面推进，2005年全市取消了农业税，农民负担持续减轻。社会保障制度改革取得重大进展。财政、金融体制改革等继续深化。

（五）县域经济发展取得重大突破。

2005年全市8个县（市、区）的GDP、规模工业增加值、固定资产投资、消费品零售总额等多项经济指标均超过全省平均增长水平。清新、英德、佛冈、连州等4个县（市）的GDP增长在全省67个县市中囊括前四名，其中清新县连续两年名列第一。清城、清新、英德、佛冈、连州等5个县（市、区）的GDP增长均创1988年以来新高。连山、连南、阳山的经济增长也创近八年来新高。英德、清新、清城等3个县（市、区）GDP均达到50亿元以上，其中英德突破70亿元。清城、清新、佛冈等3个县（区）的工业总产值均达到60亿元以上，其中清城区突破100亿元大关。五年来，佛冈县工业产值增长5.3倍，清城区增长4.8倍，清新县增长3.4倍，英德市增长2.3倍，连州市增长2.2倍。镇级经济规模迅速壮大，初步形成了一批工业强镇，2005年工业总产值突破亿元的镇（街）达到35个，比2000年增加19个。达到10亿元以上的乡镇13个，其中清城区龙塘镇61.36亿元，佛冈县石角镇45.02亿元，清新县太和镇41.71亿元。

（六）人民生活水平达到总体小康。

2005年，人民生活水平已总体达到小康。其中人均GDP达到1100美元，比2000年增加513美元。在岗职工平均工资18321元，比上年增长15.6%，比2000年翻了一番多，五年年均增长16.1%。农民人均纯收入3618元，比上年增长8%，五年年均增长6.1%。市区居民人均可支配收入达到9325元，翻了一番。城镇居民人均居住面积达到26.4平方米，增加12.2平方米。城镇居民恩格尔系数44%，人均预期寿命达到75岁，农村初级卫生保健基本合格县比重100%。以上各项指标均达到或超过小康标准，开始向建设全面小康社会目标迈进。

（七）创品牌工作大步迈进。

积极实施名牌带动战略，争创区域品牌和产品名牌。“十五”时期至今，我市先后被国家和省授予“中国优秀旅游城市”、“广东省文明城市”、“广东省双拥模范城”以及“中国漂流之乡”、“中国温泉之乡”、 “中国龙舟之乡” 等多项称号；英德市被授予“中国英石之乡”、“中国红茶之乡”、“中国麻竹笋之乡”称号；阳山县被授予“四驱之都•越野之城”称号；清新县被授予“广东省旅游强县”称号，地区知名度大大提高。积极鼓励和推动企业创名牌。新亚光电缆有限公司的新亚牌6—35KV高联乙烯电力电缆产品被评为“中国名牌产品”，并和远光电缆实业有限公司的“光”牌系列电力电缆产品一起被评为“国家免检产品”。“林中宝”、“凤中皇”、“徐其修凉茶”、远光电缆、南华水泥、广英水泥、连州东陂林泉腊味、金鑫纸业、华能制药等9家企业商标被评为“广东省著名商标”，这9家企业和南山水泥厂的产品被评为“广东省名牌产品”。连州东陂林泉腊味通过国家地理标志产品审核。实现了我市“中国名牌产品”、“国家免检产品”、“广东省著名商标”和“国家地理标志产品”零的突破。

（八）民主法制建设明显加强。

市政府认真执行市人大及其常委会各项决议并向其报告工作，坚持向人民政协通报情况。认真办理人大议案、建议和政协提案及群众来信，五年来，共办理人大建议507件，政协提案和来信782件。自觉接受监督，先后有4名副市长和10名政府组成部门负责人接受人大评议。密切联系人民团体，积极接受社会监督。大力开展法制教育，“四五”普法任务全面完成。政务公开、村务公开和厂务公开全面推进，依法治市工作取得明显成效。基层民主政治建设取得新进展。

（九）社会各项事业全面进步。

教育实力明显增强。大力推行素质教育和信息技术教育，全部中学和乡镇中心小学以及有条件的农村完全小学开设了信息课程，教育整体水平和质量进一步提高。巩固“普九”成果，适龄儿童入学率达99.4%，初中入学率达97.1%，15周岁初等教育完成率和17周岁初级中等教育完成率分别达到99.4%和96.4%；老区小学改造任务全面完成，中小学薄弱学校改造成效显著；民办教育得到快速发展；职业教育发展迅猛，2005年，全市职业技术学校在校生2.68万人，比2000年增长1.4倍；做大做强职业教育品牌，清远市技工学校被评为国家级重点技工学校，清远市第一职业技术学校被评为国家级重点职业技术学校，创建了清远职业技术学院。

文化体育事业蓬勃发展。积极推进文化大市建设，市博物馆、图书馆等一批文体设施陆续建成。与中央电视台联合成功举办了“心连心”慰问演出，开展广场文化艺术节、“北江情—清远旅游体育文化艺术节”、“火红五月•激情清远”、连州国际摄影节等系列大型文艺演出和展览活动。广播电视、新闻出版事业有新发展。圆满完成了全国篮球赛、漂流赛、龙舟赛、钓鱼赛和四驱越野车赛等一系列赛事的承办任务。成功举办了第二、三届全市运动会。“十五”期间，清远籍运动员在世界性赛事上获2枚金牌，在全国赛事上获金牌10枚。

卫生工作卓有成效。城乡两级公共卫生设施建设显著加强，医疗条件明显改善，行业监管力度不断加大，药品、医疗器械采购制度进一步完善。农村卫生工作不断加强，新型农村合作医疗保障制度进一步健全，基本实现县级统筹，2005年，全市参加农村合作医疗人数为173.7万人，占农村总人口的56.2%。

劳动就业工作取得新成绩，社会保障体系不断完善。劳动者自主择业、市场引导就业格局基本形成。五年累计输出劳动力46.51万人次，新增就业岗位17.45万个，培训下岗失业人员2.31万人次，职业技能培训12.7万人次，国企下岗失业人员再就业3.9万人次。城镇登记失业率控制在3.53％以下。养老、失业、生育、医疗和工伤保险参保人员分别达到27.17万人、13万人、8.27万人、17.38万人和15.15万人。实现了社会保险市级统筹，各项社保基金积累达到11.37亿元，确保国有企业离退休人员基本养老金和国有企业下岗职工基本生活费按时足额发放。社会救助工作有效开展。城乡最低生活保障已全面实现应保尽保。

各项事业全面发展。“十五”期间，全市新增省高新技术企业25家、省民营科技企业24家；科技兴农成效显著，信息化建设迈上新水平，建成了电子政务中心平台，开通了清远市政府网。科普工作有效推进，清新县获“全国科普示范县”称号。知识产权保护工作和减灾防震工作得到加强。环境和资源保护力度不断加大。建成了市区和英德污水处理厂；全市主要江河水质良好，饮用水源水质达标率为100%；城市空气质量达到国家环境质量二类区标准，城市声环境和生态环境质量保持稳定；林业生态工程稳步推进，建成国家级自然保护区3个、省级自然保护区4个，饮用水源保护区15925公顷，全市森林覆盖率达到66.8%；生态县创建工作取得“开门红”，连山县已被省批准为“广东省林业生态县”；清远成为全省第二个全国水土保持示范城市。人口与计划生育工作成绩显著，2005年人口自然增长率为5.14‰，比2000年下降1.06个千分点，被省评为人口与计划生育工作先进单位，清城区连续八年被评为先进单位。圆满完成了第五次人口普查和第一次经济普查工作。民族宗教工作得到新发展，被省评为“民族团结进步”先进集体。公民道德教育活动、未成年人思想道德教育活动和创建文明单位、文明窗口活动扎实开展。严厉打击“法轮功”等邪教组织；有效预防和妥善处置群体性事件；始终保持高压态势，严厉打击各类刑事犯罪；社会治安防控体系建设进一步加强，人民群众安全感进一步增强。市场秩序进一步规范，打击假冒伪劣商品工作受到省政府表彰，非法传销活动得到有效遏制。审计监督力度明显加大。安全生产专项整治成效明显，重大事故隐患和重大危险源监控工作得到加强，食品卫生安全和特种设备安全实现零事故。外事、侨务、对台、人事、妇女儿童、扶贫、人防、移民、武装、气象、档案、方志、残联等各项工作有新发展。

（十）政府施政能力不断增强。

我们始终坚持以人为本，围绕“建设一流的班子、带出一流的队伍、培养一流的工作作风、增创一流的工作业绩、树立一流的政府形象”的目标，严格按照“诚信为民、勤政廉洁、务实高效”的要求，切实转变政府职能，积极推进行风评议，大力整顿机关作风，着力加强自身建设，政府施政能力明显提高。

依法行政水平进一步提高。认真实施国务院《全面推进依法行政实施纲要》，全面修订《清远市人民政府工作规则》。行政执法责任制进一步健全，治理公路“三乱”和中小学乱收费成果得到巩固，廉政建设和反腐败力度持续加大，纠正损害群众利益的不正之风取得明显成效。

政府调控宏观经济能力进一步提高。建立了宏观经济预报和监测制度，充分发挥物价、统计、审计、工商、质监、药监、银监等部门作用，确保经济的快速健康发展。

政府管理社会能力进一步提高。成功战胜“非典”疫情，有效应对禽流感，妥善处理重大污染事件，以铁的手腕依法炸封关闭51对证照不全的小煤矿，社会公共卫生和安全体系进一步完善。切实强化信访工作，加大调解力度，积极清偿征地款、工程款和农民工工资，依法规范城市拆迁行为，各类矛盾纠纷和信访案件得到妥善处置，群众集体上访事件明显减少，群体性事件明显下降。各项社会管理初步迈入科学化、法制化和程序化轨道。

政府服务能力进一步提高。建立了行政服务中心，完成了市政府规范性文件的清理工作，进一步减少了办事环节，提高了服务水平。

各位代表，过去五年，我市国民经济和社会发展取得了令人振奋的成绩，这是我们坚持邓小平理论和“三个代表”重要思想，全面贯彻落实科学发展观的结果，是省委、省政府和市委正确领导的结果，是各级人民代表大会及其常务委员会和人民政协监督支持的结果，是全市人民同心同德、奋发图强、开拓创新、聚精会神搞建设，一心一意谋发展的结果，是港澳台同胞、海外侨胞和社会各界关心支持的结果，是兄弟地区尤其是挂扶单位大力帮助的结果。在此，我代表市人民政府向全市广大工人、农民、知识分子、干部职工、驻清人民解放军、武警、消防官兵、人民警察和各民主党派、人民团体、社会各界人士表示崇高的敬意！向长期关心和支持清远建设的兄弟地区、香港和澳门特别行政区同胞、台湾同胞、海外侨胞和外籍友人，表示衷心的感谢！

各位代表，“十五”时期，我们之所以能取得令人满意的业绩，关键是我们在工作中认认真真做到了“六个始终坚持”：一是始终坚持以全面落实科学发展观作为各项工作的思想指南。深刻领会科学发展观的实质内涵，把握“五个统筹”，在加快城市建设的同时，注重加快农业农村的发展；在加快南部和东部地区发展的同时，注重推动北部山区的发展；在加快经济发展的同时，注重推进社会各项事业的发展；在加快经济社会发展的同时，注重环境保护工作，追求人与自然的和谐发展；在加大对外开放力度、做好招商引资工作的同时，注重加快民营企业的发展，确保我市经济社会的全面、协调、可持续发展。二是始终坚持以改革创新作为攻坚破难的强大动力。我们以解放思想、创新观念为先导，为加快发展注入了强大的思想动力；通过因地制宜，创新发展战略、创新政策，最大限度地激发了发展潜力；通过各项改革，进一步解放了生产力；通过整合各项资源，为加快发展开拓了新的空间。三是始终坚持以实施“三化一园”发展战略作为加快发展的根本途径。通过大力实施“三化一园”发展战略，我市经济社会出现了跨越式的发展，地区由冷变热，发展由慢变快，排位由后变中，初步实现了经济起飞的目标。四是始终坚持以招商引资作为经济工作的重中之重。各级政府把招商引资作为经济工作的头等大事，着力营造“洼地效应”，创新招商引资方式，调整利益分配制度，下移招商引资主体，大力开展招商引资，引进了一大批知名大企业，促进了经济的快速发展。五是始终坚持以发展特色经济作为增强经济竞争力的突破口。充分利用我市的区位优势和环境优势，引导客商投资我市具有比较优势的产业，千方百计把自身优势转化为生产力，促进了我市建材陶瓷、电力、食品加工、再生资源利用、旅游、房地产等行业迅速发展，规模经济水平不断提高，全国最大的水泥生产基地、全国十大循环经济示范基地之一的华清循环经济示范基地等初现端倪。六是始终坚持以以人为本、关注民生作为执政为民的最高宗旨。我们始终将人民群众根本利益作为一切工作的出发点和落脚点，一心一意为人民谋福祉，千方百计解决社会弱势群体的生产生活问题。认真抓好农村安居工程、基础教育网络工程、农村饮用水工程、城乡水利防灾减灾工程、小型水库除险加固和机电排灌工程、革命老区实现“三通”工程、农村医保和疾病预防控制中心主体建设工程、低收入群体基本生活保障体系建设工程、文化建设工程、治污保洁工程等“十项民心工程”建设，取得了预期效果。

我们也清醒地看到，当前我市经济社会发展存在的困难和问题，突出表现在六个方面：一是我市经济发展水平还比较低，经济总量还比较小，具有较强市场竞争力的规模企业和名牌产品不多，经济结构仍需优化。二是土地、能源、适用劳动力等生产要素的供给压力增大，转变经济增长方式日益迫切。三是农村经济发展整体滞后，农民增收压力较大，“三农”工作任重道远。四是区域经济发展差距有所扩大，北部山区加快发展任务艰巨。五是基础设施还不能满足经济快速协调发展需要，建设步伐仍需加快。环境保护工作欠帐较多，产业化发展步伐缓慢。六是文化、教育、卫生、体育事业还不能满足人民群众日益增长的需要，发展力度有待加大。我们必须高度重视存在问题和不足，采取有力措施逐步加以解决，为顺利完成“十一五”各项目标任务创造更加良好的发展环境。

二、“十一五”时期的奋斗目标和主要任务

“十一五”时期，是我市全面建设小康社会的关键时期。我市既面临难得的发展机遇，又面临严峻的挑战，但总体上有利条件更多。国际、国内区域合作不断扩大和加强，产业资本加速向低成本地区转移。国家和省都向欠发达地区实施更积极的发展扶持政策。我市通过18年的建设，已积累了一定的发展基础，全市经济社会进入了新一轮的快速增长期。但与此同时，国际市场竞争越来越激烈，国内兄弟地区迅猛发展，而制约我市发展的一些深层次矛盾和问题又尚未从根本上得到解决。为此，我们必须认清形势，抓住机遇，乘势而上，进一步转变观念，创新发展模式，增强发展后劲，推动我市经济社会发展实现新的跨越。

“十一五”时期我市经济社会发展的总体目标是：全市生产总值年均增长18%，到2010年，实现GDP比2000年翻两番，人均GDP达到或超过全国平均水平；地方财政一般预算收入年均增长20%；单位生产总值能源消耗比“十五”期末降低15%以上；社会各项事业取得更大发展，精神文明与民主法制建设明显加强，人民生活水平显著提高。

要实现“十一五”目标，必须以邓小平理论和“三个代表”重要思想为指导，以科学发展观统领经济社会发展全局，正确处理好“六个关系”：一是正确处理好发展速度与发展质量的关系，加快经济增长方式转变。要在保持较快发展，做大经济总量的同时，不断提高经济社会发展的质量和效益，实现速度、质量与效益，经济、社会与环境的全面协调发展。二是正确处理好外源型经济与内源型经济的关系，促进内外源型经济协调发展。要在提高对外开放水平，继续加强招商引资的同时，坚定不移地发展民营经济，壮大内源型经济，不断增强自我发展能力。三是正确处理好引进技术与自主创新的关系，逐步增强自主创新能力。要在坚持引进先进技术的基础上，把增强自主创新能力作为科学发展的战略基点和调整产业结构、转变增长方式的中心环节，千方百计提高引进、消化、吸收和再创新水平。四是正确处理好城市化与建设新农村的关系，促进城市和农村同步发展。要在进一步加快城市化建设进程的同时，全面推进社会主义新农村建设，大力改善城乡面貌。五是正确处理好经济发展与社会发展的关系，推进和谐清远发展。要在发展经济、增加物质财富的同时，不断增加社会各项事业的投入，促进社会各项事业的繁荣进步，构建和谐清远。六是正确处理好改革、发展、稳定的关系，努力为科学发展提供保障。要把改革的力度、发展的速度和社会可承受的程度统一起来，在保持社会稳定中推进改革与发展，通过改革和发展促进社会稳定。

要完成“十一五”目标，必须继续深入实施“三化一园”发展战略，做到八个“扎扎实实”：

（一）扎扎实实做大经济总量。加快经济发展步伐,继续争当山区经济发展排头兵。走新型工业化道路，集中力量发展规模经济、循环经济，做大做强支柱产业;加快农业产业化步伐,推进农业规模化经营，提升农业发展水平;大力发展旅游业和现代服务业，改造提升传统服务业，不断壮大第三产业；继续采取有效措施促进投资、消费和出口的快速增长。

（二）扎扎实实提高发展质量。积极发展技术密集型产业，广泛应用高新技术和先进适用技术改造提升传统产业。鼓励、支持企业建立技术创新体系。不断优化产业布局和产业结构，重点建设水泥、陶瓷、有色金属和农副产品加工、纺织制衣、制鞋皮革、电子家电、汽车配件和化工等产业基地，延伸产业链条。大力发展效益农业、特色农业。深入实施品牌战略，不断推进国民经济和社会信息化。

（三）扎扎实实增强发展活力。继续深化各项改革，不断健全和完善现代市场体系。加大招商引资力度，增强承接发达地区产业转移能力；优化利用外资和出口贸易结构；大力发展民营经济，重视发展本地规模大、技术含量高、产业辐射带动能力强的民营企业，推动外源型和内源型经济相互促进、共同发展。实施“人才强市”战略，加大人才引进和培养力度，不断优化人才发展环境，为我市经济社会发展提供人才资源保障。

（四）扎扎实实加强基础设施建设。在完成第二个“八路一桥”工程的基础上，要谋划建设第三个“八路一桥”项目；抓好北江航道整治和清远客运南北站场的建设，在交通基础设施上率先融入珠三角。进一步加大水利设施建设力度，增强防洪减灾、水资源保障和水环境保护能力。加快能源建设和电网改造，合理调整能源布局结构和供给结构。

（五）扎扎实实推进城乡和区域协调发展。加大城市和中心城镇基础设施配套建设力度，不断完善服务功能，增强聚集能力。大力推进社会主义新农村建设。以更大力度推进南部地区工业化进程，促进南部地区追赶珠三角发展水平；推动东部地区快速崛起，形成特色产业优势；加大力度支持北部地区振兴经济，尤其要用办特区的理念进一步扶持少数民族地区经济社会发展。

（六）扎扎实实推进大文化建设。不断增加对科技、教育、文化、体育、卫生等领域的投入，进一步加强大文化设施建设。优先发展教育，大力发展科技、文化产业，繁荣体育事业，完善医疗卫生体系，丰富人民群众的精神文化生活，创建国家文明城市。

（七）扎扎实实解决人民切身利益问题。继续实行积极的就业政策，努力扩大就业，完善社会保障体系。维护妇女儿童合法权益。高度关注弱势群体，不断完善社会救助体系。加大帮扶力度，切实帮助困难群众解决生产生活问题。

（八）扎扎实实推进和谐清远建设。加强民主法制建设，认真开展“五五”普法教育，全面推进依法治市。加强政府自身建设，提高依法行政水平。整顿和规范市场秩序，营造法治、诚信、公平、文明的市场环境。完善社会纠纷调处机制，妥善处理各方面利益关系，维护和促进社会公平。做好国防动员和民兵预备役工作，深入开展“双拥”活动，进一步密切军政军民关系。建立健全社会预警体系和应急救援机制，提高公共突发事件应对处理能力。加强环境保护和生态建设。全面落实安全生产、社会治安综合治理和维护稳定的各项措施，努力形成社会安定、环境安全、百姓安宁的良好局面。

各位代表，“十一五”蓝图已经绘就，我们对实现“十一五”发展目标充满信心。坚信在省委、省政府和市委的正确领导下，经过全市人民的不懈努力，一个经济繁荣、社会和谐的新清远必将在南粤大地崛起！

三、2006年工作安排

2006年是实施“十一五”规划的开局之年，也是全面落实科学发展观、在新的起点上实现发展新跨越的关键一年。今年市政府工作的指导思想是：坚持以邓小平理论和“三个代表”重要思想为指导，以科学发展观统揽工作全局，认真贯彻落实十六届五中、六中全会、中央经济工作会议和省委九届七次、八次全会和市委四届八次全会精神，按照“统筹年”、“跨越年”、“和谐年”的总体要求，深层次推进“三化一园”发展战略的实施，统筹经济、政治、文化和社会的协调发展，努力实现主要经济指标增幅继续走在全省前列，和谐清远建设上新水平，为“十一五”时期的新跨越奠定坚实基础。

今年全市经济社会发展的主要预期目标是:生产总值增长18%，其中，一、二、三产业分别增长4%、31%和14%；单位生产总值能源消耗比上年下降3%；地方财政一般预算收入增长20%，其中工商税收增长22%；全社会固定资产投资增长25%；实际利用外资增长33%；社会消费品零售总额增长16%；外贸出口总额增长34%；居民消费品价格指数控制在3%左右；在岗职工工资增长10%，农民人均纯收入增长6%；城镇登记失业率控制在3.5%以内；人口自然增长率控制在7‰以内。

为实现今年经济社会发展预期目标，必须突出做好以下工作：

（一）加快发展规模经济，提升产业发展水平。

大力发展规模经济，着力做好“四培育一提升”：一是培育规模园区，为工业发展提供更加广阔的平台。对现有工业园区进行整合、延伸和提高，进一步完善工业园区的路、水、电、通讯等基础设施及生活配套设施建设。以园区吸引项目，以项目支撑园区，加快产业转移工业园、循环经济产业园等工业园区建设，不断扩大工业园区规模，以规模园区带动规模工业的发展。不断提升工业园区的管理水平，打造一批上规模、上档次的品牌园区，以吸引更多大企业来落户，吸引更多上下游企业来配套。二是培育规模产业，打造区域名牌。立足自身优势，加快工业布局调整步伐，因势利导，做大做强建材、陶瓷、能源、生物医药、电子家电、纺织服装、汽车配件、金属制品、农副产品加工、制鞋皮革等十大产业，加强对产业发展的引导和规划，形成有特色、有规模的产业集群，提升区域经济综合竞争力。抓住机遇，发挥优势，规划建设上规模的中药材生产、加工基地。三是培育规模企业，增强辐射带动能力。加强对行业龙头企业的培育，支持企业进一步增资扩产，延伸产业链条，不断发展壮大。引导企业走出去，广泛参与区域经贸合作与交流，拓展发展空间。四是培育规模经济区域，打造“工业强县（市、区）”。集中力量，发挥优势，努力打造一批工业产值在100 亿元以上的县（市、区）、50 亿元以上的镇、10 亿元以上的村，加快奠定规模区域的发展格局。五是提升企业自主创新能力，打造优势品牌。引导企业加快推进技术进步，鼓励有条件的企业建立产品研发技术中心，自主开发创新。大力推进“质量兴市”，实施名牌带动战略，增强品牌意识，开展品牌经营，支持和鼓励企业争创国家、省级品牌，提高企业和产品市场竞争力。

（二）提高招商引资水平，增强经济发展后劲。

一是提升招商引资质量。继续实行招商引资主体下移，发挥县、镇两级在招商引资中的作用。广泛动员社会力量参与招商，进一步扩大招商引资规模。实行有选择招商，下大力气引进规模大、技术含量高、污染低、效益好、带动能力强的项目。不断创新招商引资方式、方法，将引进项目与引进技术、管理和人才结合起来，不仅要重视引进企业，更要重视引进产业链。二是抓好签约项目的跟踪落实。强化招商引资考核机制，把招商引资的预期目标和项目落实继续列入各级政府、有关职能部门政绩考核范围。对已经签约的重点项目，要加强协调，合力推进，确保项目按期履约开工和顺利投产。着力抓好全市120个重点项目建设进程，尤其是要推进海螺水泥二期工程、台湾水泥、金德管业等一批投资规模大、产业关联度高、支撑带动能力强的大型项目建设和达产，做到投产一批、开工一批、储备一批，不断增强经济发展后劲。三是大力改善招商引资环境。进一步强化服务意识，提高办事效率。继续完善和落实招商引资优惠政策及奖励措施，营造新的“洼地效应”，不断增强招商引资吸引力。

（三）大力发展县域经济，促进区域协调发展。

抓住各级扶持和发展县域经济的机遇，以争创经济强县（市、区）为动力，借助外力，启动内力，促进县域经济健康快速发展，实现市、县 （市、区）、镇三轮驱动。一是要在发展速度上实现新的跨越。在基础设施、资源型项目规划建设上向连阳地区倾斜，以办特区的理念，加大少数民族地区的政策、产业扶持与基础设施建设力度，尤其是要加大投资80亿元的清连一级公路改高速工程建设力度，改善北部地区交通环境，促进北部地区加快发展步伐；加快东部地区特色经济发展步伐，打造规模产业基地；加快南部地区高新技术产业发展，做大做强生物医药、建材陶瓷、轻工电子等龙头产业，形成规模经济效应，加快与珠三角的产业融合。二是要做大做强镇域经济。鼓励有条件的镇依托资源和产业优势，集中精力做大做强规模产业，打造一批各具特色、具有较强竞争力的经济强镇。三是加快民营经济发展步伐。进一步落实发展民营经济的政策措施，消除民营经济发展的各种障碍。强化政府引导、协调、规范和扶持作用，推进中小企业综合服务机构建设，逐步建立和完善综合服务、信用担保、人才培训、信息咨询四大服务平台。引导民营企业进一步规范管理，加快建立自主创新机制，开展创品牌活动，提高技术水平和竞争力。支持具有一定规模的民营企业通过兼并、收购、联合等方式做大做强，发展成为主业突出、市场竞争能力强的大集团大公司。

（四）着力加强“三农”工作，推动社会主义新农村建设。

推进社会主义新农村建设，以此为重点带动“三农”工作上新水平。一是抓好新农村建设的总体规划和试点工作。加强调研，科学编制全市新农村建设总体规划，确定农村发展新格局。抓好全市30个新农村建设试点。积极与高等院校合作开展新农村建设试点工作。二是继续大力调整农业和农村经济结构，切实增加农民收入。以清远鸡、三元杂猪等为重点，抓好品种改良、规模化、标准化养殖，提高畜牧业在农业总产值中的比重。继续推进“八个五”工程建设，完成年度建设任务；积极做好省、市“一乡一品”项目的实施，加快发展中草药种植业，加大家禽、蔬菜、水果、有机稻等13个优势农产品基地建设力度；做大做强麻鸡、乌鬃鹅、水晶梨、黑皮冬瓜、砂糖桔等一批知名品牌产业规模；进一步培育和扶持农业龙头企业、农业中介服务组织、农产品营销大户和经纪人；大力发展现代农业，继续实施“种子工程”、“植保工程”、“畜禽良种工程”和“农业机械化示范工程”，加快农业科技创新和成果转化；完善农业标准体系和农产品质量监测，大力发展无公害食品、绿色食品和有机食品生产基地，抓好无公害农产品认证。三是大力发展农村公共事业。进一步完善新型农村合作医疗制度，参加合作医疗人数要达到农村总人口的60%以上；积极开展以乡镇为单位的农村初级卫生保健工作；继续实施农村安居工程，重点抓好省安排的8条村和市安排的3000户安居工程建设；四是加强农村基础设施建设和生态保护。加大农村公路建设力度，加快镇通行政村公路路面硬底化建设。完成5000户农村沼气池建设；抓好农村改水、改厕工作，加大村容村貌整治力度，力争创建2-3个省级卫生镇、一批省级卫生村。五是落实党在农村的各项政策。继续稳定、完善和强化各项支农政策，深化和巩固农村税费改革成果，从今年秋季开始实施农村免费义务教育。加强农业生产资料市场监管，严禁向农民乱收费、搭车收费，进一步减轻农民负担。六是倡导文明新风，培育新型农民。破除陋习，移风易俗，倡导科学文明健康的生活方式。大力弘扬“勤劳、自强、互助”精神，培育“有文化、懂技术、会经营”的新型农民。七是进一步加快农村劳动力转移步伐。大力引导农民向二、三产业转移，抓好基层劳务输出中介服务。大力实施“广东省百万农村青年技能培训工程”，加强农民实用技术和就业技能培训，完成培训5万人、转移4万人的任务，拓宽农民增收渠道。

（五）加快发展第三产业，促进经济全面繁荣。

一是加快发展旅游业。做好旅游发展总体规划，促进旅游有序发展。抓好旅游资源整合与深度开发，抓好“五大品牌”、“五条热线”重点旅游项目建设。完善景区配套服务设施，提高接待能力和服务水平，争创国家A级景区。大力开拓商务旅游、城郊旅游、“农家乐”旅游等新项目。加大宣传力度，认真办好飞碟、漂流、龙舟、越野等四大国际国内体育赛事，进一步提高清远知名度。连州、英德和阳山要扎实开展创“中国优秀旅游城市”和“广东省旅游强县”活动。二是加快城乡市场建设步伐。抓好专业市场规划建设，特别是农副产品、建材陶瓷、汽车配件、五金家电等专业批发市场建设，扶持英德建设全国最大的观赏石集散市场，加快形成一批规模化、集约化、专业化批发市场。培育和扶持一批物流龙头企业，大力发展连锁经营、物流配送、电子商务等现代流通业，逐步把我市建设成沟通泛珠三角的南北物流走廊。三是加快拓宽消费市场。鼓励和支持有条件的企业走出去，开拓境外市场。进一步优化外贸出口结构，提高外贸出口质量和水平。实施万村千乡市场工程试点，完善农村市场网络，加快消费市场设施建设，优化消费环境，扩大消费规模。大力发展咨询、保险、会计、设计装饰、中介、会展经济等现代服务业，引导形成汽车、房地产、教育、娱乐、休闲、体育健身等消费热点，提升消费水平。

（六）加大投入力度，进一步完善基础设施。

加强水利、交通、能源、通信等基础设施建设。一是加强水利设施建设。抓好 62宗小型水库除险加固工程、一批农村饮水工程、机电排灌工程、饮水安全等工程建设，全面完成城乡水利防灾减灾工程，增强防灾减灾能力。二是加强交通基础设施建设。加快清连高速公路改造、北江大桥扩建工程等第二个“八路一桥”建设进程，谋划好第三个“八路一桥”重点工程项目。协助做好漳昆、二广高速和武广铁路客运专线建设工作。三是加强电源电网建设。继续做好挖掘小水电发电潜能工作，积极争取连南煤矸石发电厂、粤连电厂三期改造等项目的立项，着力抓好核电站、抽水蓄能电站项目的跟踪落实。进一步完善电网结构，抓好500千伏清远输变电工程和220千伏朗新（英德）、燕河（清城）等输变电工程建设，增强电源输送能力。四是加强通信设施建设。

加强城市基础设施建设。完善城市总体发展规划，做活“山、水、绿、文、人”文章，塑造城市特色。加快飞来湖、大燕湖建设步伐，争取清远北江水利枢纽工程早日动工，建设中国“湖城”。抓好市区骨干道路的改造以及美化、亮化、绿化、净化、黑底化工程规划与建设，把桥南路—桥北路—清和大道打造成市区商业中轴线；抓紧新市区断头路的连接，加快横荷、人民西路、迳口三个道路出口的整治和建设。加大城中村的整治与改造力度。加快城市无障碍设施建设，营造关怀残疾人的良好环境，提升城市文明水平。各县（市、区）也要抓好城市中轴线、水轴线以及城镇骨干道路、出口、城市景观等的规划建设。加快省确定的22个中心镇的规划与建设。

（七）加大改革创新力度，增强新的发展动力。

今年要重点推进以下六项改革：一是加快完成国有企业改革任务。多渠道筹集国有企业改革专项资金，妥善解决职工安置问题，全面完成国有企业改革攻坚任务。二是扎实推进农村综合改革。积极推进农村集体资产和财务监管机制改革。进一步规范农村土地承包经营权流转，加强农村土地管理。抓好撤并镇村的配套完善工作，巩固并镇并村成果。三是建立健全多层次的利益共同体。进一步改革和完善市中心区域利益共同体；建立市与县（市、区）利益共同体，实行大项目不论地域，全部冠清远名称，市、县两级利益分成；建立市与部门利益共同体，正确处理收支两条线与统收统支的关系。调动各方面积极性，发挥各级在促进经济发展中的能动作用。四是深化行政体制改革。进一步推进政府机构改革，加快政府职能转变，继续深化行政审批制度改革，以建设服务型政府为重点，全面履行经济调节、市场监管、社会管理和公共服务职能。五是进一步整合资源。继续加快卫生资源的优化重组，推进医疗体制改革，做大做强卫生产业；继续加大教育资源整合力度，创新办学体制，鼓励社会资本办教育，扩大重点高中办学规模，大力发展高中阶段教育。六是加快户籍制度改革。落实各项户籍管理制度改革措施，促进城镇人口有序聚集，加快城市规模扩张。

（八）加强环境保护和生态建设，促进可持续发展。

一是大力发展循环经济。加强资源综合利用，在工业园区积极开展循环经济试点工作，加快推进国家第一批循环经济试点项目—清远华清再生资源示范基地、国家废旧电子信息产品回收处理示范基地建设，探索发展循环经济的有效模式，逐步完善再生资源回收利用体系。二是大力倡导节约生产。广泛开展节能节水节地节材活动，形成健康文明、节俭合理的消费模式，努力建设节约型社会。三是加大环境保护力度。做好环境保护规划，大力发展环保产业，加强水源保护，加快大燕河水污染整治。在水泥、陶瓷、冶金、有色金属、建材、化工、电力等重点行业推广清洁生产。及时解决关系群众切身利益的突出环境污染问题。综合治理工业污染、农业农村环境污染和城市污染，坚决治理重点污染企业，加快高新区、旧城污水处理厂建设和生活垃圾及危险废物处理设施建设。加强建设项目环境管理，认真把好环保准入关，从源头上防治污染和保护生态。加大植树造林力度，加强对土地、森林、矿产、自然保护区的生态保护与管理。四是加强生态环境建设。积极开展生态示范区创建活动，抓好市区自然生态区（“市肺”工程）的规划建设，推进城市绿化工作，创建省级园林城市，加快林业生态县创建步伐，进一步改善人居环境。

（九）大力增收节支，认真做好财税金融工作。

一是继续加强财政工作。深化部门预算、政府采购和收支两条线管理等改革，扩大市级国库集中支付改革试点范围并在县级全面推进，建立县级财政增收节支奖励机制。抓紧做好党政机关经营性资产清理工作，努力挖掘潜力，提高非税收入质量。强化预算约束，严格控制一般性支出，严格控制财政供养人员规模，最大限度发挥公共财政作用。优化支出结构，提高财政对基础设施、社会保障、科技教育、文化卫生和环境保护等公共事业的保障力度。逐步完善财政监督体系和绩效预算评价体系。二是强化税收征管。坚持依法治税，加强重点税源管理，大力组织税收。加强税务信息化建设，提高纳税服务水平。严厉打击各种偷逃骗税行为，促进应收尽收。三是维护金融稳定。防范和化解金融风险，不断改善金融生态环境，打造诚信清远；积极发挥金融对经济发展的支撑作用，切实加大金融机构对我市的信贷支持力度；深化农村信用社改革，推进金融产品创新，解决中小企业和民营企业融资难问题。

（十）全面发展各项社会事业，促进经济社会协调发展。

优先发展教育事业。继续巩固和扩大“普九”义务教育成果，加快高中、职业技术教育和成人教育发展，抓好市一中实验中学、华侨中学新校区、清新县一中新校区、市高级技工学校等重点学校建设，推动清远职业技术学院二期工程建设，抓好7所骨干示范中等职业学校建设。推进教育强镇创建工作，力争创建2-3个市级教育强镇和1个省级教育强镇。

大力发展科技事业。推动企业技术创新，争取省新认定一批高新技术企业和民营科技企业。积极推进省、市确定的9个专业镇技术创新试点工作。建立健全农村科技、信息推广网络。加强科普宣传，努力提高全民科技意识和水平。

进一步繁荣文化事业。继续加强公民道德教育、未成年人思想道德教育和文明社区、文明单位、文明窗口创建活动；积极开展文化下乡、文化扶贫活动，组织形式多样的群众性文化活动。大力发展文化产业，培育文化娱乐市场，抓紧新文化广场立项和市青少年宫、市少儿图书馆及县级“三馆”建设。抓好“盘王节”等文化品牌的申报工作。加强文学艺术创作和广播电视、新闻出版工作，繁荣哲学社会科学，不断满足人民群众日益增长的精神文化需要。

提高医疗卫生水平。建立和完善各项管理和监督制约机制，降低医疗成本和医疗收费。加快市人民医院新院建设，完成市疾控中心、市血站、市慢病院疗养分院等医疗卫生设施建设，逐步完善医疗救治、应急和疾病控制体系。继续完善城市社区卫生体系，落实农村初级医疗保障制度，不断提高公共卫生服务水平。加强中医药管理，推进中医药事业发展。强化药品、食品和公共场所卫生监督管理，保障人民群众身体健康和生命安全。

加快发展体育事业。抓好第十二届省运会及省民族运动会的备战与参赛工作。巩固和提升“中国漂流之乡”、“中国龙舟之乡”的品牌效应，积极做好“中国足球训练之乡”的申报工作，推动全民健身活动深入开展。

巩固人口与计划生育工作。进一步推行计划生育层级动态管理，建立计划生育利益导向机制，落实人口与计划生育目标管理责任制。加大综合治理出生人口性别比升高力度，加快推进人口现代化。

推进社会各项事业全面发展。加强少数民族工作，促进民族团结；进一步做好“双拥”和优抚工作；全面推进和谐社区建设。继续做好统计、外事、侨务、对台、人事、妇女儿童、残联、宗教、扶贫、移民、武装、人防、气象、地震、档案、方志等各项工作。

（十一）切实关注民生，努力构建和谐清远。

做好就业再就业工作。实施积极的就业政策，拓宽就业渠道，增加就业岗位，优化就业环境，加强就业服务。抓好农村富余劳动力转移就业和失业职工再就业工作，加大对零就业家庭、“4050”人员的就业和再就业援助。加强职业技能培训，加快市高级技工学校“二次创业”进程，组织实施省“三年五万”新技师培养工程，加强职业技能鉴定。加快健全劳动关系调整机制，维护劳动者合法权益。

完善社会保障体系。继续加大社会保险的扩面征缴、清欠力度，完善养老、医疗、失业、工伤和生育保险制度，进一步扩大社会保障覆盖面，不断完善社会保障体系。完善社会保险市级统筹工作机制。统筹协调社会利益关系，建立社会公平保障体系。

切实关心困难群众的生产生活。高度关注弱势群体，完善城乡低收入困难群众社会救助体系，切实帮助困难群众解决“一保五难”问题。加大劳动监察力度，积极开展清理拖欠征地补偿款、农民工工资和工程欠款工作。创新扶贫开发思路，大力推进科技、教育和智力扶贫，完成培训和新增输出贫困劳动力6000人任务。继续实施“十百千万”干部驻村扶贫，切实帮助驻点村解决实际问题，加快经济发展。加快发展慈善事业和社会福利事业，确保城乡弱势群体得到及时有效救助。加大助学扶志工作力度，帮助困难学生完成高中阶段教育。

以铁的手腕抓好安全生产工作。加大安全生产宣传教育力度，提高全民安全意识。落实安全生产责任制，健全安全生产监管体制，严格安全执法。继续巩固煤矿安全专项整治成果，突出抓好交通、非煤矿山、危险化学品、人员密集场所消防安全等专项整治，确保烟花爆竹生产企业在半年内全面退出，防止重大安全事故发生，力争各类安全事故死亡人数同比下降3%。

切实维护社会稳定。继续整顿和规范市场秩序，依法严厉打击假冒伪劣、价格欺诈等不法行为，营造安全可靠的消费环境。加强社会治安综合治理，开展专项斗争，严厉打击各种黑恶势力、严重刑事犯罪和“两抢”、“两盗”等多发性犯罪；坚决扫除“黄赌毒”、传销和“六合彩”赌博；加快社会治安防控体系建设，开展平安和谐社区创建活动，提高社会治安综合治理水平。改革创新信访工作机制，及时排查发现各种社会矛盾，认真调处化解各种纠纷，使人民群众的合理诉求得到妥善处理。

（十二）加强政府自身建设，推动政府效能全面提升。

推进政府工作民主化进程。坚持向人大及其常委会报告工作和向人民政协通报情况的制度，自觉接受人大及其常委会的法律监督和人民政协的民主监督。认真落实人大及其常委会的决议，切实办好人大代表议案、建议和政协提案。进一步推进政务公开、村务公开和厂务公开。完善重大事项民主协商、决策参与和民主监督制度。启动“五五”普法教育，推进依法治市。坚持依法行政，深入贯彻实施《全面推进依法行政实施纲要》，全面推行行政执法责任制，坚持依法决策、依法办事、依法处理问题。加强公共应急平台建设，不断完善突发公共事件应急处置机制，提高预防和妥善处置群体性事件能力。继续改进机关工作作风，提高工作效率。坚持“四个服务”，大力推进政府管理创新和制度创新，建立部门协调配合机制，切实提高服务水平。加强公务员队伍建设，认真贯彻落实《公务员法》，完善公务员管理制度，加强督查考核，做到从严治政，赏罚分明，促进勤政廉政，建设高素质的公务员队伍。坚持教育、制度、监督并重，加大反腐败的工作力度，树立“为民、务实、清廉”的政府形象。

各位代表,“十五”取得的巨大成就，鼓舞了我们的斗志，增强了我们的信心；“十一五”规划纲要绘制的美好蓝图，更坚定了我们争当山区发展排头兵的决心。让我们更加紧密团结在以胡锦涛为总书记的党中央周围，高举邓小平理论和“三个代表”重要思想的伟大旗帜，以科学发展观统领经济社会发展全局，在市委的正确领导下，团结一致，鼓足干劲，求真务实，开拓创新，为实现“十一五”规划目标，为全面完成今年各项任务，为实现经济社会发展的新跨越起好步、开好局而努力奋斗！
